# Promoting and inhibiting: Corporate charitable donations and innovation investment under different motivation orientations——Evidence from Chinese listed companies

**DOI:** 10.1371/journal.pone.0266199

**Published:** 2022-04-13

**Authors:** Hongpeng Wang, Yang Zhang, Ming Tian, Zhenhua Wang, Yuan Ding

**Affiliations:** 1 Business School, Hohai University, Nanjing, Jiangsu, China; 2 World Water Valley Institute, Nanjing, Jiangsu, China; Universiti Pertahanan Nasional Malaysia, MALAYSIA

## Abstract

Corporate charitable donations under different motivations will have different effects on innovation investment through different action paths, which provides a new perspective to solve the inconsistency of existing research results. Based on the resource-dependent and principal-agent theories, this paper compares and discusses the relationship between charitable donations and innovation investment under different motivations. Using 2008 ~2019 relevant data of listed companies as research samples, a mixed regression model is established for the hypothesis test, and further examines the state-ownership of its moderating role. The results show that the altruistic motivation-oriented corporate donations have a significant inverted u-shaped effect on innovation investment. The tool motivation-oriented corporate donations have a significant U-shaped effect on innovation investment. Moreover, it is further found that for ST (Special Treatment) corporates with the risk of delisting in the tool motivation-oriented charitable donations type, the corporate charitable donations have a significant negative effect on innovation investment. State-ownership can enhance the inverted U-shaped relationship between altruistic motivation-oriented corporate donations and innovation investment but weaken the U-shaped relationship between tool motivation-oriented corporate donations and innovation investment.

## 1. Introduction

Corporate charitable donations refer to the enterprise that donates a certain amount of money, in-kind, or services to the needy to alleviate the shortage of public services and products, which is the most representative corporate social responsibility behavior [1]. However, with the increasingly competitive business environment, some charitable donations are no longer motivated solely by gratuitous altruism. They are more focused on the instrumental function of corporate charitable donations, trying to establish or maintain a competitive advantage through charitable donations. For example, Alibaba Group’s charitable donations for the prevention and treatment of COVID-19 are more focused on the targeted use of innovation investments. Alibaba Group donated funds to support medical research through contracts with several top research institutions, including the Chinese Academy of Sciences and the Zhong Nanshan Medical Foundation. This approach to donations has allowed Alibaba Group to share the pressure of the Chinese government’s epidemic response while helping to bring together significant innovative resources of its own to build a competitive advantage in the healthcare sector quickly. In increasing environmental uncertainty, corporate charitable donations have been given more strategic significance in the current context. On the one hand, enterprises must actively undertake corresponding social responsibilities to create an ideal external survival space [2, 3]. On the other hand, they must maintain stable innovation investment to obtain the ability to survive and develop [4]. Therefore, it is essential to clarify further the relationship between charitable donations and innovation investment for the survival and development of enterprises in the current environment.

Corporate innovation investment, which mainly includes labor wages and R&D investment, can strengthen the degree of product differentiation and thus create a corporate competitive advantage. Stable innovation investment is considered a critical way for companies to survive and grow in the fierce business competition [4]. From the perspective of resources, both corporate charitable donations and innovation investment take up a large number of corporate resources, so whether corporate charitable donations inhibit innovation investments is still the focus of academic debates [1, 5]. Scholars who hold the " promotion" view focus on corporate charitable donations’ external resource acquisition function based on theories such as resource dependency theory and stakeholder theory. They believed that corporate innovation activities could not be carried out without the support of external resources, and corporate charitable donations can help corporate obtain essential innovation resources to form a "resource effect" to promote innovation investment [6–9]. In contrast, Scholars with an " inhibiting" perspective argued a " crowding out effect" of charitable donations on innovation investment [4, 6]. Based on principal-agent theory, they focused on the impact of charitable donations on internal resource allocation [4]. They argued that corporate agency problems could induce excessive corporate donations behavior and thus inhibit corporate innovation investment, such as agents chasing personal reputation through charitable donations. In fact, charitable donations affect firms’ access to external innovation resources and affect the allocation and utilization of internal resources, so it is necessary to explore the relationship between charitable donations and innovation investment from a more comprehensive perspective. To solve the problem of inconsistent research results, scholars had begun to focus their research on the motivation of charitable donations, trying to explore the influence mechanism of charitable donations on innovation investment from a more systematic perspective. They had confirmed that charitable donations of firms with different motivations have different effects on innovation through different mechanisms [10–13]. That provides a good entry point for further exploring the impact of corporate charitable donations on innovation investment.

Depending on the underlying assumptions, existing research on the motivation of charitable donations mainly includes tool-motivation and altruistic-motivation. The tool-motivation view is based on classical economics assumptions (depending on the purpose of tool-motivation can be further subdivided into strategic motivation, political motivation, and motivation of management utility), and believed that the only goal of enterprises is to maximize profits. Charitable donations are only seen as an essential tool for achieving that goal [6, 14]. Charitable donations under this motivation aim to gain social reputation, reduce litigation risk, reduce government regulation, and obtain compensation from external resources [15, 16], which can create a "resource effect" to promote innovation investment by obtaining external resources [7, 8]. In contrast to tool-motivation, altruistic-motivation is based on the assumption of the social-ethical attributes of enterprises and believes that corporate charitable donations are purely altruistic acts without expecting returns [17]. Especially in the context of a socialist market economy with Chinese characteristics and the Chinese culture of "wealthy people helping the world," charitable donations guided by altruistic-motivation have been given the critical mission of promoting the harmonious development of society and pursuing the spirit of selfless giving without any expectation of return [18, 19]. However, most of the existing studies on the motivation of charitable donations are based on a single dimension, such as irregularities and the excessive pursuit of media attention [20, 21], which makes it challenging to grasp the characteristics of charitable donations under different motivations comprehensively. As a result, most existing studies have focused on charitable donations with tool-motivation, but there is a lack of in-depth research on charitable donations with altruistic-motivation [22], making it challenging to explore the mechanism of charitable donations on innovation investment comprehensively. Therefore, it is necessary to summarize the typical characteristics of different charitable donations’ motivations from multiple dimensions. To build a more systematic research framework, explore the relationship between charitable donations and innovation investment under different motivations more comprehensively, and further answer the question of whether charitable donations have a promoting or inhibiting effect on innovation investment.

To address the lack of relevant research, this study draws on psychological measures of motivation to summarize the typical characteristics of different motivations for donations in two dimensions: "purpose" and "behavior" [23], in order to build a research framework for classifying different types of motivations for donations. And based on the integrated resource dependence theory and principal-agent theory, we compare and analyze the role relationship between corporate charitable donations and corporate innovation investment under different donation motivation orientation, so as to clarify the relationship between charitable donations and innovation investment. In addition, the ownership of enterprise in China’s socialist market economy is typically dualistic, and the incentives and constraints faced by state-owned enterprises and the business environment are quite different from those of non-state-owned enterprises [24]. This research question focuses on corporate charitable donations in the Chinese market context, and thus it is necessary to further explore how differences in corporate ownership affect the relationship between corporate charitable donations and innovation investment under different motivations. In summary, this study examines the relationship between the role of charitable donations on innovation investment under different donation-motivations, using Chinese listed companies from 2008 to 2019 as the research sample. We also focused on the characteristics of the Chinese market and further examined the regulatory role of corporate ownership on the relationship between the two to provide corresponding theoretical guidance for healthy corporate development.

By doing so, we found the following: (1) The altruistic motivation-oriented corporate donations have a significant inverted u-shaped effect on innovation investment. (2) The tool motivation-oriented corporate donations have a significant U-shaped effect on innovation investment; Moreover, it is further found that for ST (Special Treatment) corporates with the risk of delisting in the tool motivation-oriented charitable donations type, the corporate charitable donations have a significant negative effect on innovation investment. (3) State-ownership can enhance the inverted U-shaped relationship between altruistic motivation-oriented corporate donations and innovation investment but weaken the U-shaped relationship between tool motivation-oriented corporate donations and innovation investment.

The contributions of this work are as follows:(1)This study constructed a research framework for classifying different types of charitable donation motivation by psychological motivation measurement methods, which provides a new entry point for a more systematic exploration of the relationship between charitable donations and innovation investment. (2) By integrating resource dependence theory and agency theory, a new theoretical framework is provided to explore the nonlinear relationship between charitable donations to innovation investments. (3) The findings enriches the knowledge in the relationship between corporate charitable donations and innovation investment.

The remainder of this work is organized as follows. Section 2 briefly describes the literature that provide the basis for this work, construct a research framework for classifying types of motivations for charitable donations, and propose research hypothesis; Section 3 introduces the methodology used in the research; Section 4 demonstrates and analyze the results of the research. Robustness test and endogeneity test were involved. Section 5 shows further discussion about the results; Section 6 summarizes the main findings of the study and concludes with the managerial implications for further research.

## 2. Literature review and hypothesis development

### 2.1 Corporate charitable donations and innovation investment

The discussion on the relationship between the role of corporate charitable donations on enterprise innovation investment is essentially a question of how to allocate the limited resources within the organization between charitable donations and innovation investment [25, 26]. Existing research has fully confirmed that corporate charitable donations can have a "resource effect" positive effect or "crowding out effect" negative effect on innovation investment under different conditions [4, 27, 28]. First of all, the viewpoint of resource dependence theory points out that the resources of external knowledge and capital are essential to the innovation activities of enterprises [29]. In the process of obtaining innovative resources from the outside, the external stakeholders of enterprises often play the key roles of resource provider, regulator and so on, which can effectively help enterprises to obtain the resources needed for innovation [27, 30]. Thus, the "resource effect" of promoting enterprise innovation investment is formed. While emphasizing the maintenance and protection of good relationships between enterprises and stakeholders, resource dependence theory also points out that organizations’ dependence on external resources must respond to or meet the needs of external stakeholders who provide resources [31], but the limited internal resources of the organization require enterprises to handle the "exchange" relationship with the resources of the external stakeholders of the organization carefully and reasonably, and to carry out charitable giving activities rationally. In modern enterprises, agents have the task of balancing the interests of many stakeholders. Because agents have self-interest motivation [32], such as pursuit of reputation, social status and other reasons, to produce excessive donation behavior [33], charitable donations tend to crowd out innovative resources phenomenon [4].

Through a review of the existing literature, it can be found that, firstly, resource dependence theory emphasizes that firms cannot invest in innovation without the support of external resources, and corporate charitable donations can help firms obtain the resources needed for innovation investment by helping them build good relationships with key external stakeholders. The agency theory, on the other hand, focuses on the impact of agency problems on the internal resource allocation of enterprises, and believes that the agency problems of enterprises will lead to excessive donations, which will have a negative impact on innovation investment by competing with enterprises’ innovation activities for resources. The former is based on the perspective of external resource acquisition, while the latter focuses on the internal resource allocation and utilization of the firm. Therefore, in view of the complexity of the relationship between charitable donations and innovation investment, this study argues that it is necessary to integrate resource dependence theory and agency theory to provide a more systematic theoretical basis for the study of the influence mechanism of charitable donations on innovation investment. Secondly, under the joint action of “resource effect” brought by charitable donations and “crowding out effect” brought by agency problems in the process of charitable donations, it is bound to make the relationship between charitable donations and innovation investment show a complex nonlinear relationship. Therefore, it is necessary to distinguish charitable donations according to different donation motivations in order to avoid the interference of complex factors on the research results.

### 2.2 Distinguishing between different types of corporate charitable donations’ motivations

**Fig 1:**
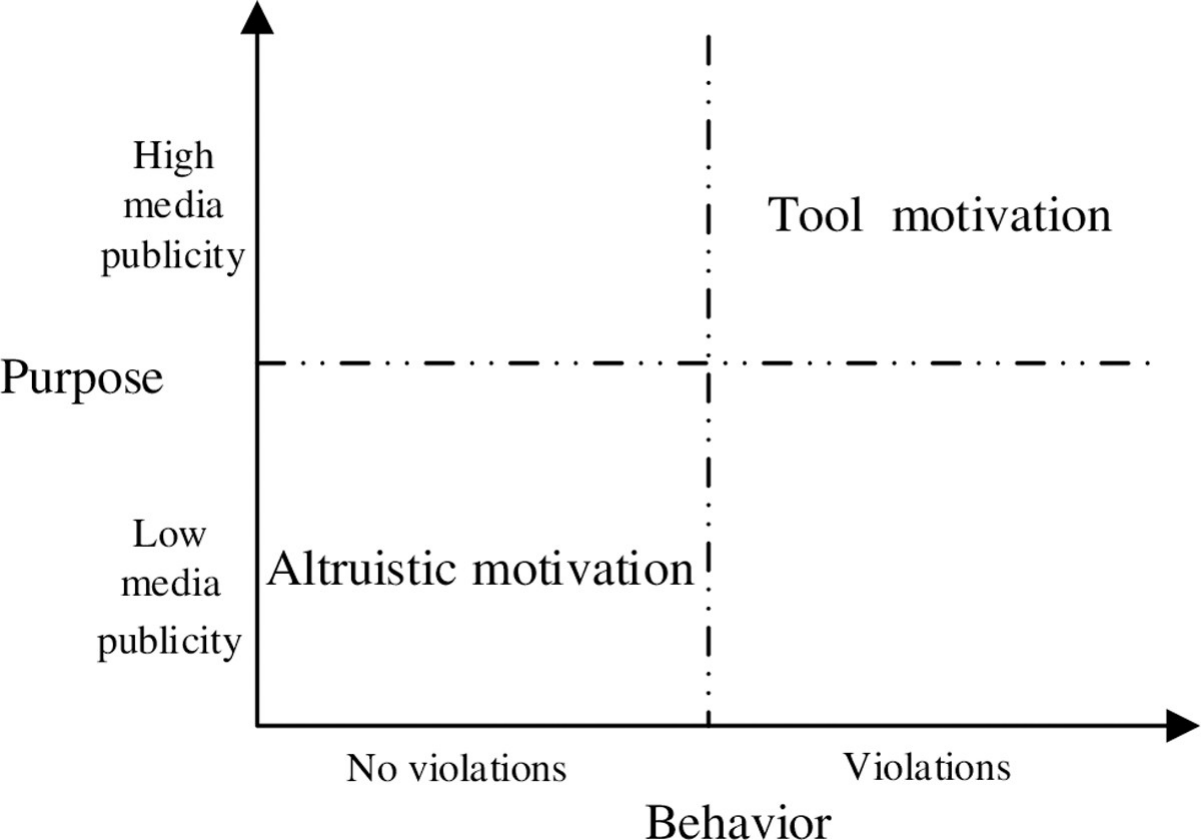
The framework for classifying types of corporate charitable donations motivation.

It is often difficult to directly measure the motivations of corporate charitable donations, so we draw on psychological behavioral motivation measurement methods [23] to summarize the typical characteristics of different types of charitable donations’ motivations in two dimensions: "purpose" and "behavior", and thus build an analytical framework to classify different types of charitable donations’ motivations (Fig 1). Firstly, from the perspective of donation "behavior" dimension, whether there are corporate violations behind the charitable donation behavior is one of the important objective basis to distinguish between " tool" or "altruistic" motivations [15, 34]. According to Carroll’s definition of corporate philanthropy, corporate philanthropy is an altruistic behavior that does not directly expect economic returns and voluntarily donates funds, materials, and other resources to support social welfare undertakings [35]. Compared with altruistic motivation-oriented charitable donations, tool motivation lies in concealing or "whitewashing" corporate irregularities rather than purely doing good. In this regard, Li X [20] confirmed through research that companies would voluntarily increase charitable donations to achieve the purpose of concealing or ’whitewashing’ when irregularities occur. Secondly, from the perspective of the "purpose" dimension of charitable donations, companies that make charitable donations under the tool motivation focus more on the promotion of their "charitable image" than the altruistic motivation. The reason is that under the guidance of tool charitable donation motivation, enterprises need to publicize the positive image of enterprises through the media to achieve non- altruistic purposes such as transmitting good financial conditions to the outside world and conceal violations [17, 21], for example, the KANGMEI Pharmaceutical Co., LTD, which has won the ’China Charity Award’ for many consecutive years, still invested nearly 60 million yuan((More than 30 percent of its R&D investment that year) in charitable donations even in 2018, which was deeply affected by the ’financial fraud’. It should be pointed out that enterprises implementing altruistic charitable donations do not use charitable donations as a gimmick to publicize.

Notably, it should be noted that while companies that implement "altruistic" charitable donations do not use charitable donations as a gimmick for publicity, they can have a beneficial impact on their social networks. The reason is that, firstly, corporate charitable donations are a kind of pure good behavior independent of the usual marketing behavior of enterprises, which is an essential part of its feedback society, fulfilling corporate social responsibility. Secondly, in the Chinese cultural context, a low-key approach to charity and a focused product attitude will give people a good image of "integrity." It will be more conducive to establishing or maintaining a stable social network [36].

Based on the "The framework for classifying types of corporate charitable donations motivation" constructed in this study, corporate charitable donations are classified into altruistic motivation-oriented charitable donations and tool motivation-oriented charitable donations. We define charitable donations that neither have irregularities nor actively seek excessive charitable media attention as altruistic-motivated charitable donations. Accordingly, we define charitable donations with corporate irregularities and actively seek media exposure as tool motivation-oriented charitable donations.

### 2.3 Altruistic motivation-oriented corporate charitable donations and innovation investment

First of all, Carroll believes that Corporate social responsibility(CSR) has a hierarchical nature, including economic, legal, ethical, and charitable levels. Charitable donations are at the highest level, and only after companies have achieved their economic, legal, and ethical goals will they proceed to charitable donations [35]. Companies that make charitable donations with altruistic motivation do not have a rigid need to conceal corporate irregularities. Therefore, companies that make charitable donations with this motivation often have already accomplished their economic, legal, and ethical goals at this stage. Their charitable donations can use redundant corporate funds [20] without crowding out resources for innovation investments. Secondly, as a kind of prosocial behavior, charitable donations can help enterprises to maintain and strengthen the external links of the organization, significantly improve the quality and efficiency of resource exchange between enterprises and many stakeholders to obtain the critical resources needed for enterprise innovation investment [9], form a "resource effect," and promote enterprise innovation investment. The social capital required for enterprise innovation increases with charitable donations [37]. When the level of corporate charitable donations is low, although the corresponding charitable investment has been paid, it has not yet reached the social-psychological expectation, so the social reputation generated cannot attract enough external resources to promote enterprise innovation, and the enterprise innovation performance is low. With the improvement of charitable donations, the influence of enterprises increases, helping enterprises and external subjects to establish a more comprehensive social network, attracting more external resources to promote the improvement of enterprise innovation level [16], such as establishing a good relationship with the government helps enterprises to obtain more preferential policies, which is convenient for the acquisition of scientific and technological resources, and then helps enterprises to invest in innovation.

**Fig 2:**
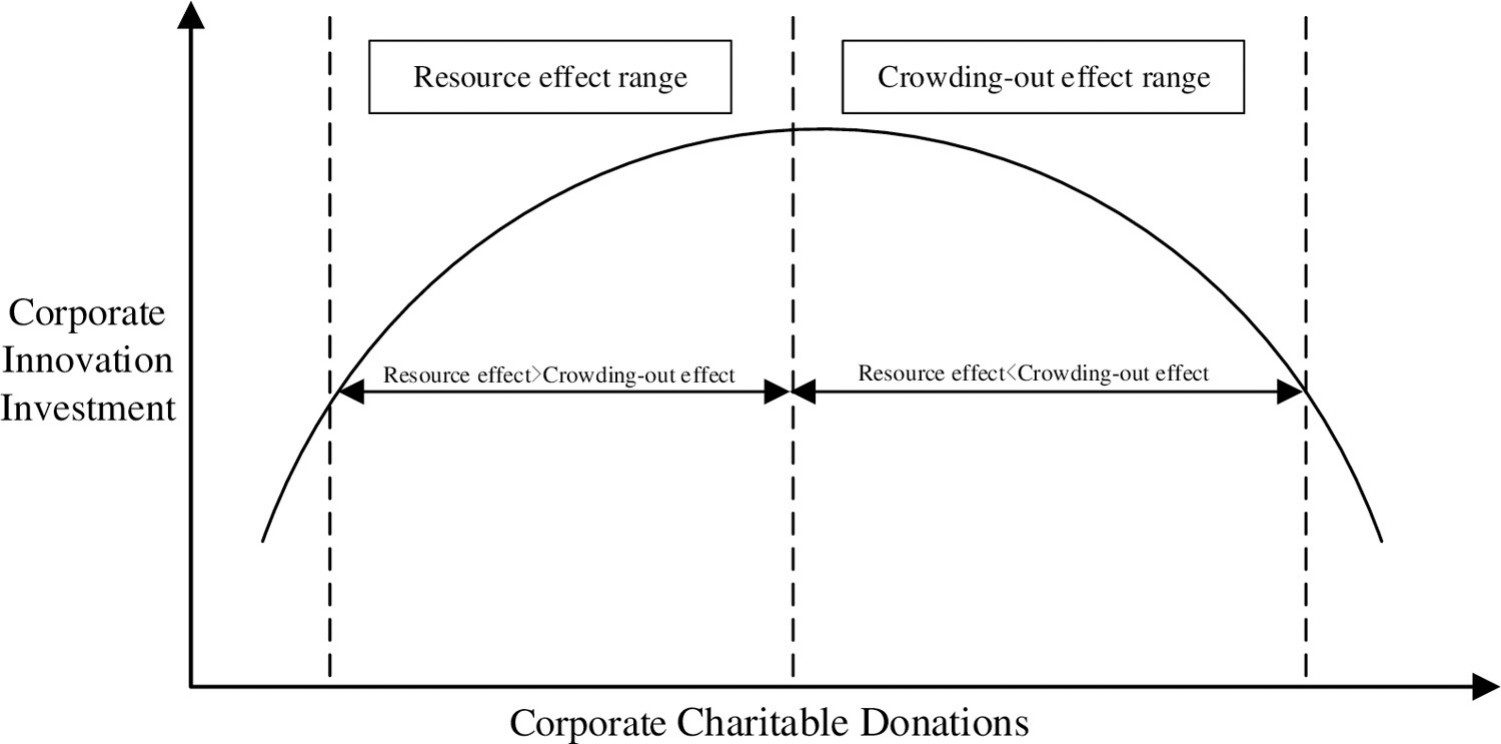
Mechanism of altruistic motivation oriented charitable donations on innovation investment.

However, charitable donations are not the more, the better for business development. When the donation investment exceeds a specific limit, such as exceeding the limit of organizational redundancy resources, high donation investment costs will gradually offset the “resource effect” to promote enterprise innovation and even occupy enterprise innovation resources, which has a negative impact on enterprise innovation investment [29]. Meanwhile, according to principal-agent theory, charitable giving is affected by agency problems even when it is driven by altruistic motivation because of widespread information asymmetry [38]. Corporate information asymmetry inevitably generates different degrees of agency problems. This makes the process of corporate charitable donations, agents tend to pursue personal reputation, social status and other reasons to generate or exacerbate excessive donations [38–40], thus creating a "crowding out effect" on innovation investment and inhibiting the innovative investment activities of enterprises (Fig 2).

H1a: Altruistic motivation-oriented charitable donations have an inverted U-shaped relationship with corporate innovation investment.

### 2.4 Tool motivation-oriented corporate charitable donations and innovation investment

According to the resource dependence theory [41], any enterprise needs to maintain contact with the outside world to ensure the stability of the source of innovation investment [42]. The enterprise violation is essentially the destruction of the relationship between the enterprise and the external stakeholders of the organization [20]. As a typical way for enterprises to perform social rules, charitable donations can repair or maintain the relationship between the enterprise and the external stakeholders of the organization to a certain extent [43]. The usual practice is to shift the public’s attention from negative events to the performance of its social responsibility through high-profile charitable donations to resist the damage of negative information to its reputation in critical moments [21]. Therefore, for the tool type of charitable donation enterprises, their charitable donations have become a key link to maintain the stable relationship between enterprises and the external environment. Once this link breaks down, the tool type of charitable donation enterprises lose all kinds of resource support from many stakeholders and risks such as reputation loss, increased transaction costs, consumer resistance, refinancing difficulties, and even legal sanctions [43]. Thus, for such enterprises, even if enterprises obtain specific social capital through charitable donations, enterprises usually choose to use resources for “whitewashing” activities [18], which is bound to occur the ’crowding-out effect’ of charitable donations on innovation investment [4, 44].

**Fig 3:**
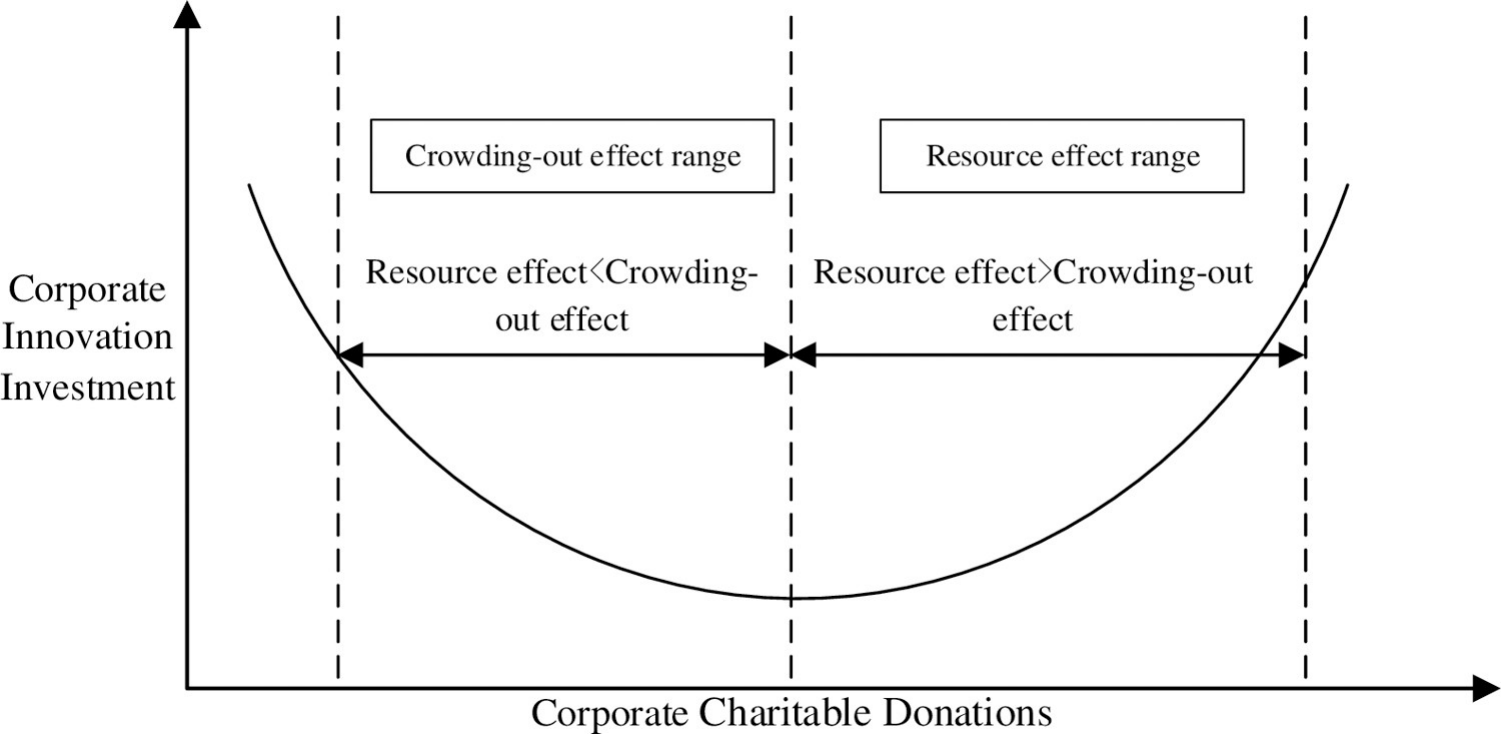
Mechanism of tool motivation oriented charitable donations on innovation investment.

Secondly, with the digestion of the negative impact of charitable donations such as ’reputation insurance’ on enterprise violations [45], the priority of charitable donations will decrease. The social capital obtained by charitable donations will be used for ’whitewash’ activities and start to pay attention to long-term development strategies such as enterprise innovation and use enterprise resources for innovation investment activities. The "resource effect" of charitable donations on innovation investment will gradually enter into force. Godfrey [46] pointed out from the perspective of the moral capital that the implementation of charitable donations by enterprises can provide prior insurance for organizations and external relations, thereby reducing the loss of corporate relational assets in the event of a crisis. Scholars such as Mino [47] had also found that corporate charitable donations help reduce the damage to corporate reputation caused by negative events in the context of product recalls. In addition, in the context of the modern enterprise management system, enterprise managers usually increase the investment in charitable donations for self-interest motivation to seek personal interests such as reputation and social status [40, 48]. However, this behavior is usually maintained within a specific limit under the role of the board of directors and other supervisory departments of enterprises, which usually does not lead to a severe crisis that threatens the survival of enterprises. When using charitable donations to occupy organizational resources for self-interest motivation to reach a specific limit, it is often warned or even eliminated by the board of directors [45], so that the crowding-out effect of corporate charitable donations on innovation investment will gradually be mitigated. After that, the resource effect brought by charitable donations on enterprises can play a role and promote enterprise innovation investment (Fig 3).

H1b: Tool motivation-oriented charitable donations have a U-shaped relationship with corporate innovation investment.

### 2.5 Moderating effect of state-ownership

Chinese economic structure presents a typical ‘duality’ feature. State-ownership enterprises(SOEs) and non-state-ownership enterprises(NSOEs) are facing different business environments that impact corporate philanthropy decisions. Compared with NSOEs, the closer relationship between SOEs and the government will exacerbate enterprises’ excessive charitable donations. That is because managers of non-state-owned property companies are more constrained by shareholders to focus on maximizing profits, usually only when expected earnings from charitable donations exceed the cost of implementation or are subject to mandatory market requirements [43]. In contrast, the managers of SOEs are more vulnerable to political objectives in business decisions. The reason is that managers (agents) of SOEs usually enjoy administrative rank, and the image of the enterprise will directly affect the image of their own officials and thus affect the promotion or not [49]. Thus, the managers of SOEs are often more likely to make excessive charitable donations for self-interest motivation in order to project and maintain their own good political image and social evaluation.

The excessive donation tendency of state-owned property rights will weaken the “resource effect” of charitable donations on innovation investment and aggravate the “crowding-out effect” of charitable donations on innovation investment. Firstly, the ‘political connection’ between SOEs and the government makes enterprise managers integrate more political identity and tasks into enterprise strategic decision-making so that enterprises take more actions to feedback the society and the government, and increase the investment of corporate charitable donations [50], thus weakening the “resource effect” of charitable donations on innovation investment; Secondly, corporate charitable donations dominated by political logic tend to produce excessive donations under the influence of multiple agency problems of SOEs’ managers, thus encroaching on the resources needed for innovation and adversely affecting innovation investment [49], thus aggravating the “crowding-out effect” of charitable donations on innovation investment.

H2a: State-ownership can enhance the inverted U-shaped relationship between altruistic motivation-oriented corporate donations and innovation investment.
H2b: State-ownership weakens the U-shaped relationship between the tool motivation-oriented corporate donations and innovation investment.

**Fig 4:**
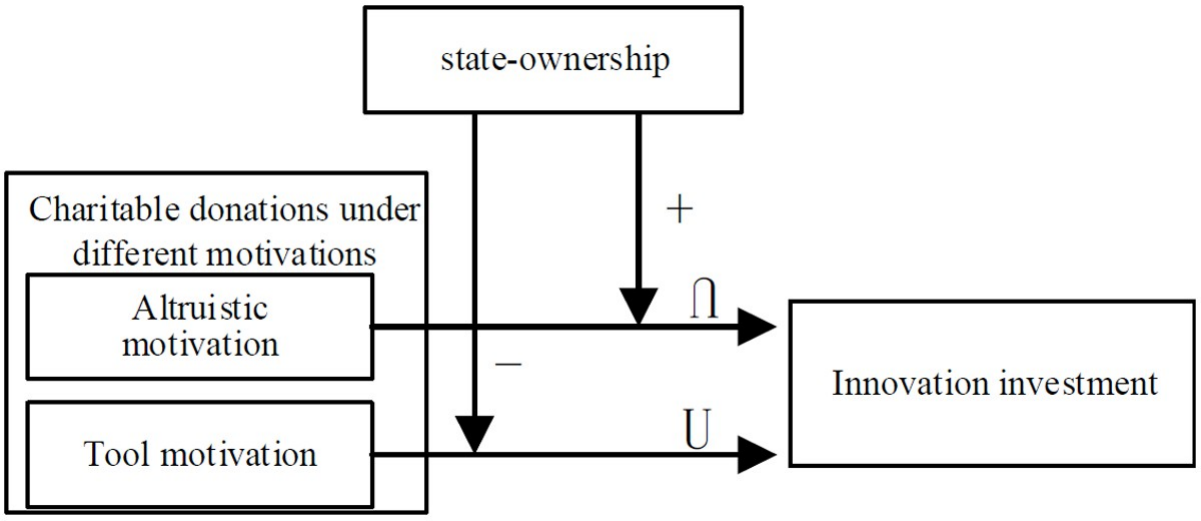
Proposed research model.

Fig 4 below shows our proposed conceptual model.

## 3. Methodology

### 3.1 Sample construction

The original sample includes all listed companies on the Shanghai and Shenzhen Stock Exchange from 2008 to 2019, and we took the following steps for data processing: (1) Eliminated data missing or does not meet the needs of this study unrelated sample data, including financial industry listed companies, ST listed companies, etc.; (2) According to the framework for classifying types of corporate charitable donations motivation (Fig 1), the sample was divided into altruistic and tool motivation-oriented charitable donations, and the specific steps are as follows: a. According to the principle of whether there are violations, the sample data are divided into two categories: violations and non-violation; b. In order to more objectively measure the subjective intention of enterprises to increase the degree of corporate publicity after the implementation of charitable donations, this study only counts the number of original enterprise positive news (As the original enterprise news is by the enterprise to bear all the media publicity costs, the content of the press release to write or review has a solid corporate subjective color. Therefore, original enterprise news can more directly reflect the purpose of the enterprise initiative to pursue media exposure) and related data reported by sample enterprises. According to the principle of whether the media publicity is greater than the median, the sample enterprises with media publicity greater than the median are divided into high publicity enterprises, and vice versa(In order to avoid the interference of data structure caused by the development of information technology such as We Media and Internet platform, this study divides media publicity according to year by year); c. Get no-violation behavior of low publicity enterprises as an altruistic motivation oriented charitable donations typical characteristics of the research sample, get violation behavior of high publicity enterprises as a tool motivation oriented charitable donations typical characteristics of the research sample.

In order to eliminate the influence of extreme values on the overall stability of the data, the continuous variables are processed by upper and lower 1% Winsorize. Finally, 1337 observation samples were obtained, including 159 ‘altruistic’ charitable donation sample enterprises, a total of 571 observation samples; There are 292 tool charitable donation sample enterprises, a total of 766 observation samples. The number of charitable donations, illegal information, R& D investment, profit level, enterprise assets, enterprise age, enterprise nature, and other related data from China Stock Market & Accounting Research Database (CSMAR) and the annual report of listed companies, network search index and media exposure, news reports and other related data from Chinese Research Data Services (CNRDS). Statistical analysis software such as EXCEL 2016 and STATA14.0 was used for data processing.

### 3.2 Variable design and measurement

#### Corporate innovation investment (RDI)

In order to reduce the impact of market value changes on data comparability, this study uses R& D intensity to measure the level of Corporate innovation investment [51], RDI = (R& D expenditure / operating income) × 100.

#### Corporate charitable donation level (DON)

In order to reduce the impact of enterprise size on the amount of corporate charitable donations, this study selects the relative donation amount to measure the level of corporate charitable donations [51], DON = (social donation amount / operating income) × 1,000.

#### State-ownership (STATE)

Referring to the research of Bai Y [52], the value of state-owned holding is 1, and that of non-state-owned holding is 0.

#### Control variable (CONTROLS)

Based on the existing research conclusions, it can be seen that the firm size, asset-liability ratio, enterprise age and industry competitiveness and other factors will have an important impact on enterprise innovation investment [48, 49, 51, 52]. Therefore, this study selected firm size (SIZE), asset-liability ratio (LEV), enterprise age (AGE) and industry competitiveness (HHI) as control variables, and controlled the industry year.

#### Endogenous control variable (E-C)

This paper focuses on the impact of charitable donations of different enterprises on innovation investment, but the two may have endogenous problems such as reverse causality. This paper introduces the variables controlling endogeneity into the model to reduce the impact of endogeneity on the research results [25]. The specific methods are as follows: The variables affecting corporate donation in year t-1 are used as independent variables, and donation in year t is used as the dependent variable for regression. Among them, AGE (p < 0.001) has a significant effect on DON for’ altruistic’ motivation-oriented corporate charitable donation samples. And SIZE (p < 0.1), HHI (p < 0.001) have significant effects on DON for tool motivation-oriented corporate charitable donation samples. For details, see Table 1. These significant variables as independent variables are regressed again, and the residual is the endogenous control variable(E-C). Variable definition as shown as Table 2.

**Table 1:** Regression results of endogenous control variables.

Altruistic motivation-oriented corporate charitable donations		Tool motivation-oriented corporate charitable donations	
Variable	Model	Variable	Model
AGE	-0.356***	SIZE	-0.003*
	(-4.32)		(-1.92)
		HHI	-0.596***
			(-3.91)
Constant	4.944***	Constant	0.690***
	(2.64)		(4.51)
Number	766	Number	566
R^2^	0.053	R^2^	0.109
Adjust-R^2^	0.026	Adjust-R^2^	0.089
F-Value	8.636**	F-Value	5.501***

^a^ ***P<0.01,** P<0.05,*P<0.1.

^b^ T-value in brackets.

**Table 2:** Definition and value method of variables.

Variable Type	Name	Sign	Variable value method and explanation
Independent Variable	corporate charitable donations	DON	continuous variable. (social donation amount / operating income) × 1,000
Dependent Variable	corporate innovation investment	RDI	continuous variable. (R& D expenditure / operating income) × 100
Regulated Variable	State-ownership	STATE	dummy argument. State holding value is 1, non-state holding value is 0
Control Variable	size of enterprise	SIZE	continuous variable. Natural logarithm of the number of employees
	assets-liability ratio	LEV	continuous variable. Total liabilities / total assets
	industry competition	HHI	continuous variable. Use (1-Herfindahl Index) to characterize industry competitiveness
	firm age	AGE	continuous variable. Enterprise age = LN (enterprise year + 1)
	Endogenous control variables	E-C	continuous variable. Residuals after DON Regression for SIZE and HHI
	Year	YEAR	Value 1 when the data falls in that year, otherwise 0
	Industry	IND	Value 1 when the data falls in the industry, otherwise 0

### 3.3 Hypothesis testing model

Since few companies disclose complete information in some years of the sample, a mixed effect model is established in the analysis due to time and sample data. Furthermore, the clustering robust standard error estimation method is used to estimate and test the model to avoid the influence of the autocorrelation between the disturbance items of the sample data in different periods.

Based on the research hypothesis, this paper set the following test model (1), (2). The formula (1) model is used to test hypothesis H1a and H1b, and the formula (2) model is used to test hypothesis H2a and H2b. Among them, *α*_0_ is the model constant, *α*_*1*_~*α*_6_ is the independent variable, the interaction between different variables and the control variable coefficient, and *ε* is the random error.


$$RDI=\alpha_{0}+\alpha_{1}DON+\alpha_{2}DON^{2}+\alpha_{3i}\sum Controls_{i}+\varepsilon$$ (1)

$$RDI=\alpha_{0}+\alpha_{1}DON+\alpha_{2}DON^{2}+\alpha_{3}STATE+\alpha_{4}DON\times STATE+\alpha_{5}DON^{2}\times STATE+\alpha_{6}\sum C\mathrm{on}trols\;i+\varepsilon$$ (2)

### 3.4 Summary statistics

In order to verify the rigor and rationality of the overall data of the selected samples, this paper uses STATA 14.0 data analysis software to descriptive statistics of the sample data, including the statistics of the mean, median, standard deviation, maximum and minimum values of independent variables, dependent variables, regulatory variables and control variables. The details as shown in Table 3. It can be found from Table 4 that the correlation coefficients are all lower than the multiple collinearity threshold of 0.5 in the empirical literature. Further analysis of variance inflation factor (VIF) for all explanatory variables shows that all sample data are far below the threshold 10 defined by empirical analysis (maximum 3.31). Therefore, it can be judged that the problem of multicollinearity is not serious, which will also be supported in regression analysis.

**Table 3:** Descriptive statistics of continuous variables.

		DON	RDI	SIZE	LEV	HHI	AGE	E-C
whole sample	N	1337	1337	1337	1337	1337	1337	1337
	Mean	3.479	0.286	8.802	0.459	0.800	2.222	-3.003
	SD	4.622	0.322	1.424	0.195	0.183	0.802	4.836
	Min	0.102	0.001	4.344	0.009	0.000	0.000	-6.700
	Max	29.240	14.712	13.165	0.869	1.00	3.332	40.732
Altruistic motivation-oriented corporate charitable donations	N	571	571	571	571	571	571	571
	Mean	3.376	0.189	8.084	0.415	0.823	2.095	-.008
	SD	3.030	0.615	1.061	0.193	0.155	0.890	1.523
	Min	0.211	0.001	4.344	0.009	0.000	0.000	-1.344
	Max	23.306	12.085	11.404	0.869	0.977	3.332	10.476
Tool motivation-oriented corporate charitable donations	N	766	766	766	766	766	766	766
	Mean	3.501	0.359	9.337	0.492	0.783	2.316	-5.220
	SD	5.494	0.906	1.426	0.190	0.200	0.715	5.237
	Min	0.102	0.197	5.900	0.056	0.000	0.000	-6.700
	Max	29.240	14.712	13.165	0.852	1.000	3.260	40.732

**Table 4:** Correlation coefficient matrix of main variables.

Altruistic motivation-oriented corporate charitable donations								
Variable	(1)	(2)	(3)	(4)	(5)	(6)	(7)	
(1)RDI	1.000							
(2)DON	0.052***	1.000						
(3)STATE	-0.225***	0.367***	1.000					
(4)SIZE	-0.256***	-0.137***	0.318***	1.000				
(5)LEV	-0.327***	-0.001**	0.293***	0.414***	1.000			
(6)HHI	0.153***	-0.068**	-0.045**	-0.043**	-0.067***	1.000		
(7)AGE	-0.239***	-0.106**	0.448***	-0.065**	0.031***	0.100**	1.000	
(8)E-C	0.076*	0.048	-0.107*	-0.026	-0.067	0.018	-0.003	1.000
Tool motivation-oriented corporate charitable donations								
(1)RDI	1.000							
(2)DON	0.177***	1.000						
(3)STATE	-0.197***	-0.107***	1.000					
(4)SIZE	-0.169***	-0.258***	0.420***	1.000				
(5)LEV	-0.198***	-0.153***	0.297***	0.409***	1.000			
(6)HHI	0.106***	0.020**	-0.027**	-0.211***	-0.063**	1.000		
(7)AGE	-0.136***	-0.065**	0.340***	0.058**	0.192***	-0.111***	1.000	
(8)E-C	-0.024*	-0.016**	0.035*	-0.001	0.016*	-0.005**	-0.045*	1.000

***P<0.01

** P<0.05

*P<0.1.

## 4. Results

### 4.1 The relationship between charitable donations and innovation investment

According to the formula (1) respectively altruistic motivation-oriented charitable donations and tool motivation-oriented charitable donations on innovation investment impact empirical analysis, regression results are listed in Table 5. For the impact of altruistic motivation-oriented charitable donations on innovation investment, Model 1 shows that control variables have a significant impact on innovation investment. In model 2, the monomial coefficient (DON) and the quadratic coefficient (DON^2^) of charitable donation level were added. The results showed that the DON coefficient was significantly greater than 0 (α = 0.558, P<0.1), and the DON^2^ coefficient was significantly less than 0 (α = -0.062, P<0.05), and the inflection point of the curve is X_1_* = 4.5. The range of DON for "altruistic" motivation is [0.211,23.306], and the inflection point of the curve is located within the range of values, so it means that "altruistic" motivated charitable donations have an inverted U-shaped relationship with corporate R&D investment, and H1a is verified.

**Table 5:** Impact of charitable donations on innovation investment.

Variable	altruistic motivation-oriented charitable donation			tool motivation-oriented charitable donation			
	Model 1	Model 2	Model 3	Model 4	Model 5	Model 6	Model 7
	RDI	RDI	RDI	RDI	RDI	RDI	RDI
DON		0.558*	0.775*		-0.059*	-0.115**	-0.012**
		(1.35)	(1.40)		(-1.79)	(-2.22)	(-2.25)
DON^2^		-0.062**	-0.172*		0.002***	0.192***	
		(-1.78)	(-1.77)		(4.98)	(5.81)	
STATE			-0.029***			-1.925**	
			(-2.68)			(-2.17)	
STATE×DON			1.289*			0.264***	
			(1.96)			(2.86)	
STATE×DON ^2^			-3.732**			-0.005***	
			(-2.29)			(-3.80)	
SIZE	-0.002**	-0.001*	-0.001	-0.004**	-0.002**	-0.002	-0.004
	(-2.26)	(-1.84)	(-0.84)	(-2.37)	(-2.28)	(-0.52)	(-1.32)
LEV	-4.037***	-3.907***	-3.798***	-4.768**	-4.453**	-3.639*	-0.004**
	(-4.10)	(-4.05)	(-3.92)	(-2.43)	(-2.36)	(-1.72)	(-2.38)
HHI	2.523***	2.486***	2.517***	2.567**	2.367**	2.442**	0.001
	(3.16)	(3.35)	(3.48)	(2.39)	(2.21)	(2.25)	(1.05)
AGE	-0.314	-0.051**	-0.031	-0.814***	-0.798***	-0.585	-0.021
	(-1.39)	(-2.09)	(-1.22)	(-2.76)	(-2.76)	(-1.49)	(-0.82)
E-C	0.120	0.112	0.094	-0.001***	-0.001***	-0.001***	
	(0.79)	(0.69)	(0.58)	(-3.31)	(-3.26)	(-2.69)	
Constant	3.726***	3.505***	4.826***	5.895***	5.913***	5.463***	0.164**
	(4.92)	(4.65)	(4.62)	(3.84)	(3.96)	(4.01)	(1.87)
Year/Industry	Control	Control	Control	Control	Control	Control	Control
F-Value	13.00***	13.67***	10.29***	9.270***	15.32***	48.88***	77.93***
N	571	571	571	766	766	766	54
R^2^	0.143	0.146	0.163	0.116	0.132	0.134	0.113
Adjust-R^2^	0.137	0.137	0.150	0.0613	0.115	0.124	0.104

^a^ ***P<0.01,** P<0.05,*P<0.1.

^b^ T-value in brackets.

Model 4 shows that control variables have a significant impact on innovation investment when it comes to the impact of tool motivation-oriented charitable donation on innovation investment. The results of Model 5 show that the monomial coefficient (DON) of the level of tool charitable donation is significantly less than 0 (α = -0.059, P<0.1), and the quadratic coefficient (DON^2^) is significantly greater than 0 (α = 0.002, P<0.01). The inflection point of the curve is X_2_* = 14.750, and the range of the DON for "tool" motivation is [0.102,29.240], and the inflection point of the curve is within the range of the values, which indicates that charitable donation for "instrumental" motivation has a U-shaped correlation with the hypothesis H1b is verified.

### 4.2 Moderating effects of state-ownership

**Fig 5:**
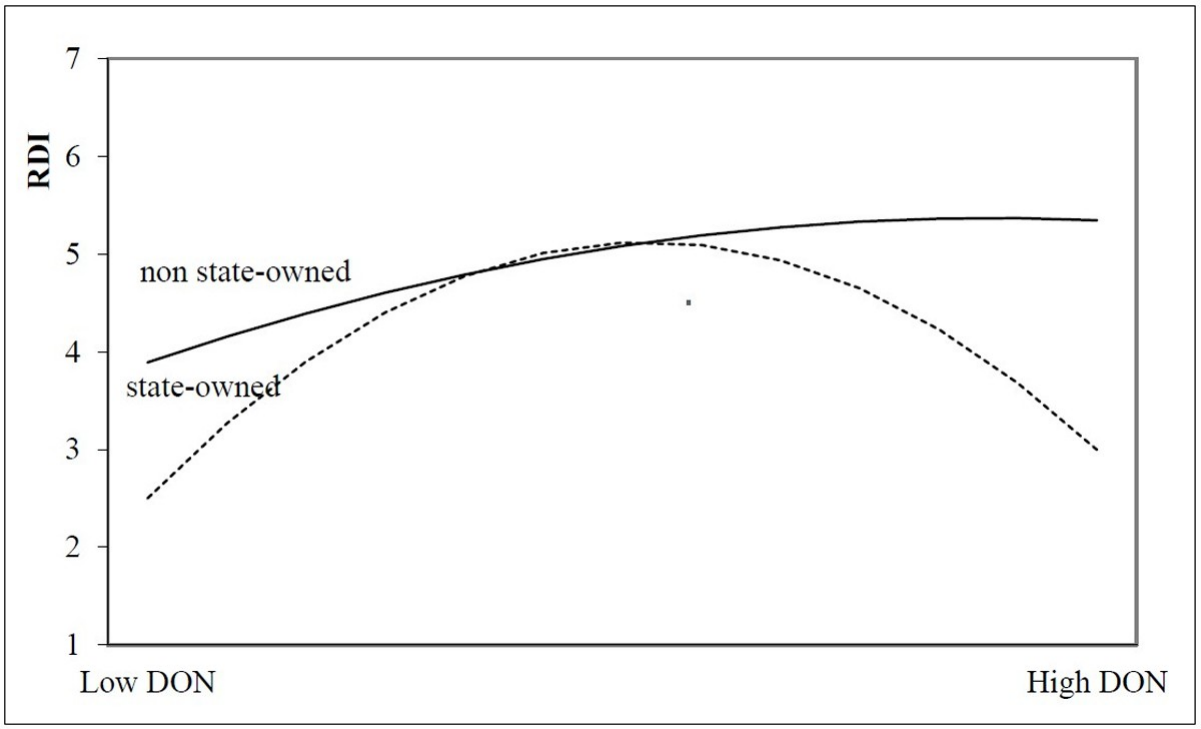
The moderating effect of state-ownership on the relationship between altruistic motivation-oriented charitable donation and innovation investment.

**Fig 6:**
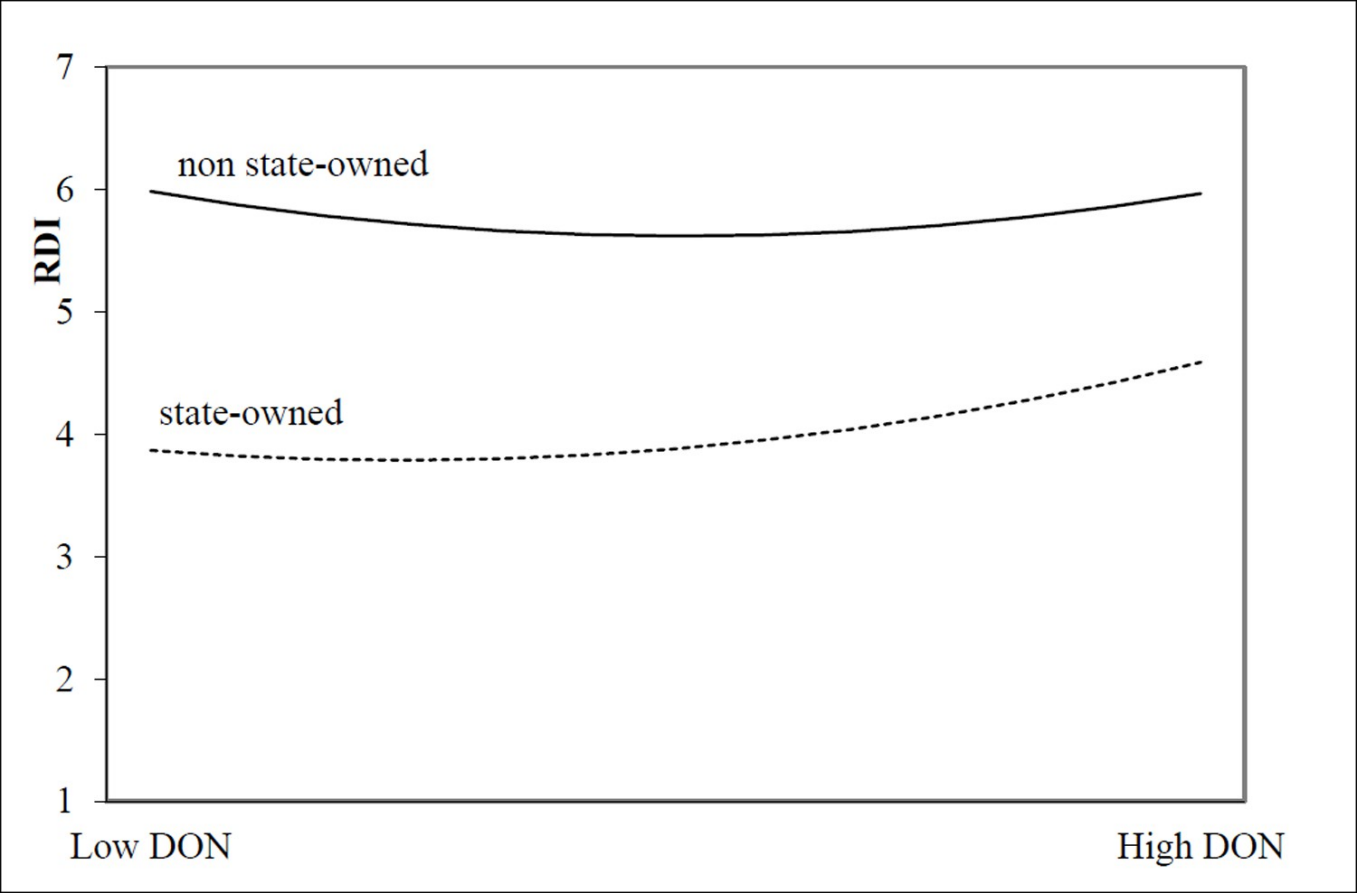
The moderating effect of state-ownership on the relationship between tool motivation-oriented charitable donation and innovation investment.

According to Eq (3), the moderating effect of state-ownership on the relationship between two types of motivation-oriented charitable donations and innovation investment is empirically analyzed, and the regression results are shown in Table 5. For altruistic motivation-oriented charitable donation sample enterprises, model 3 shows that the quadratic coefficient of state-ownership (STATE) and charitable donations is negative (α = -3.372, t<0.05), that is, state-owned property rights can enhance the inverted U-shaped relationship between charitable donations and innovation investment (as shown as Fig 5).Thus, Suppose H2a is validated. For tool motivation-oriented charitable donation sample enterprises, Model 6 shows that the quadratic coefficient of state-ownership (STATE) and the quadratic term of charitable donation is negative (α = -0.005,t<0.01), indicating that state-owned property rights can weaken the U-shaped relationship between charitable donation and innovation investment(as shown as Fig 6). Thus, Suppose H2b is validated.

### 4.3 Robustness test

In order to verify the stability of the empirical results, this paper makes robust regression by changing the measurement method of social donations. The specific methods are as follows: Referring to the measurement method of Li Sihai for charitable donations [53], DON2 = the amount of charitable donations / total assets of enterprises. The relevant results are shown in Table 6. DON2, DON2^2^ and STATE × DON2^2^have significant correlations with RDI to varying degrees respectively (at least 5% significance level test). Among them, model 8 and model 9 showed that altruistic motivation-oriented charitable donations had a significant inverted U-shaped effect on innovation investment, and state ownership had an enhanced effect on the inverted U-shaped relationship between the two; Model 10 and model 11 show that tool motivation-oriented charitable donations have a significant U-shaped relationship with innovation investment, and state ownership weakens the U-shaped relationship between them. After changing the measurement method of social donation, the empirical results have not changed significantly, indicating that the empirical results have passed the robustness test.

**Table 6:** Robustness test.

Variable	altruistic motivation-oriented charitable donation		tool motivation-oriented charitable donation	
	Mode8	Mode9	Mode10	Mode11
	RDI	RDI	RDI	RDI
DON2	0.001***	0.001*	-0.002**	-0.001**
	(15.44)	(13.45)	(-3.42)	(-2.65)
DON2^2^	-0.013***	-0.013**	0.001**	0.008**
	(-3.66)	(-3.46)	(4.54)	(7.42)
STATE		-1.918**		-1.475*
		(-2.17)		(-1.70)
STATE×DON2		-1.081**		0.118**
		(-2.88)		(2.13)
STATE×DON2 ^2^		-1.629**		-0.104***
		(2.06)		(-3.34)
SIZE	-0.002**	-0.001	-0.002**	-0.002
	(-1.97)	(-0.91)	(-2.21)	(-0.38)
LEV	-3.979***	-3.864***	-4.261**	-3.468
	(-4.09)	(-3.97)	(-2.28)	(-1.63)
HHI	-0.054**	-0.035	2.467**	2.565**
	(-2.20)	(-1.36)	(2.31)	(2.33)
AGE	2.318***	2.374***	-0.107***	-0.078
	(3.10)	(3.23)	(-2.86)	(-1.54)
Constant	3.793***	3.702***	4.939***	4.800***
	(5.00)	(4.95)	(3.62)	(3.49)
Year/Industry	Control	Control	Control	Control
F-Value	70.08***	60.99***	11.64	15.18***
N	571	571	766	766
R2	0.148	0.164	0.110	0.128
Adjust-R2	0.139	0.151	0.103	0.118

^a^ ***P<0.01,** P<0.05,*P<0.1.

^b^ T-value in brackets.

### 4.4 Endogeneity test

Based on this study, there may be two endogenous problems: On the one hand, charitable donations may have an inverse causal relationship with innovation investment; On the other hand, endogeneity may exist due to missing essential variables. This study controls the reverse causality problem by adding endogenous control variables [25] in the model construction for the first problem. For the second endogeneity problem, this study draws on the practice of Zhang Min and uses the 2SLS instrumental variable method for re-validation. Advertisement cost (ADV) is an instrumental variable to study the relationship between charitable donation and innovation investment [54]. The value method is as follows: corporate advertising expenditure / current year sales income.

Model 13 in Table 7 shows that charitable donations under altruistic motivation still have an inverted U-shaped correlation with innovation investment and pass the 1% significance level test. The results of model 15 showed that there was still a U-shaped correlation between charitable donations and innovation investment under tool motivation, and it passed the 5% significance level test. In summary, the results are still stable after considering the endogeneity problem.

**Table 7:** 2SLS endogeneity test.

Variable	altruistic motivation-oriented charitable donation		tool motivation-oriented charitable donation	
	Model 12	Model 13	Model 14	Model 15
	first stage	second stage	first stage	second stage
	DON	RDI	DON	RDI
ADV	3.316***		-0.002***	
	(-2.97)		(4.63)	
ADV ^2^	-4.592***		0.466**	
	(5.53)		(-3.57)	
DON		3.798***		-1.380**
		(2.89)		(2.55)
DON^2^		-0.359***		0.010**
		(-3.07)		(-1.89)
HHI	0.344**	-3.632***	0.866	-4.641***
	(2.08)	(-5.28)	(0.68)	(-2.88)
AGE	-0.008*	-0.011	-0.082	-0.051
	(-1.91)	(-0.72)	(-1.60)	(-1.06)
LEV	0.080	2.312***	-5.409**	-0.772
	(0.53)	(4.16)	(-2.10)	(-0.32)
SIZE	-0.000**	0.000***	-0.000***	0.000**
	(-2.08)	(5.14)	(-5.72)	(2.00)
Constant	0.273***	16.640***	7.550***	1.036
	(3.65)	(47.68)	(3.88)	(0.40)
Year/Industry	Control	Control	Control	Control
R2	0.828		0.331	
Wald chi2(6)		72.84		25.96
Prob > F	0.000	0.000	0.000	0.000
N	571	571	766	766

^a^z value in parentheses.

^b^*** p<0.01, ** p<0.05, * p<0.1.

## 5. Further discussion

According to the regression results of this study, there is a U-shaped relationship between tool motivation-oriented charitable donations and innovation investment. That is, the “crowding-out effect” of charitable donations on innovation will be improved under the “resource effect” of charitable donations and the role of corporate board supervision. However, in an actual situation, these two kinds of failure may occur. For example, KANGMEI Pharmaceutical Co., Ltd. mentioned above has not escaped the CSRC delisting warning. Therefore, since ST (Special Treatment) enterprises are excluded from the research samples, there may be a “survivor bias” problem in the research results.

In fact, for tool motivation-oriented charitable donations, there may also be a situation where charitable donations do not improve the “crowding-out effect” of innovation investment, and enterprises fall into a negative cycle of “drinking poison to stop thirst”. First of all, enterprises will increase charitable donations to cover up their illegal activities. That is, the “crowding-out effect” of charitable donations on innovation investment is no longer redundant. Secondly, although corporate charitable donations can help enterprises attract external resources through the establishment of a good image because such enterprises usually pay poor attention to product performance [18], speculative [48], and other characteristics, so often repeated violations, so that charitable donations ‘reputation insurance’ failure. In addition, even in the increasingly perfect modern enterprise management system, there is still a situation of board failure [55], which makes the excessive donation behavior of enterprise managers lose effective supervision. Finally, it is difficult to improve the “crowding-out effect” of charitable donations on innovation investment.

In order to verify this view, this study further sorts out 54 ST enterprises in Chinese listed companies from 2008 to 2019. The test model formula (3) is constructed, where *α*_0_ is the model constant, *α*_*1*_~*α*_2_ is the independent variable and the control variable coefficient, *ε* is the random error.


$$RDI=\alpha_{0}+\alpha_{1}DON+\alpha_{2i}\sum Controls_{i}+\varepsilon$$ (3)

Regression analysis is performed according to formula (3) and the results are shown in Table 5. Model 7 shows that there is a significant negative correlation between charitable donations and innovation investment (α = -0.012, P<0.05). Thus, under certain circumstances, the negative relationship between tool motivation-oriented charitable donations and innovation investment is verified.

## 6. Conclusions and managerial implications

In Chinese culture, this paper further discusses the “corporate charitable donation -innovation investment” debated by academia. The ultimate goal of this study is not to compare the difference in the value of corporate donation under different motivations, but to explore the most appropriate method for enterprise when balancing the use of charitable donations and innovation investment on enterprise resources under different donation motivation orientations. Based on the resource dependence theory and principal-agent theory, this study discusses the mechanism and boundary conditions of charitable donations on corporate innovation investment, and draws the following results.(1) altruistic motivation-oriented corporate charitable donation has a significant inverted U-shaped effect on innovation investment; U—shaped effect of tool motivation-oriented corporate charitable donation on innovation investment; for ST enterprises in the type of tool motivation oriented charitable donations, corporate charitable donations have a significant negative effect on innovation investment, that is, a complete crowding-out effect. (2) State ownership can enhance the U-shaped relationship between altruistic motivation-oriented corporate donations and innovation investment. State ownership can weaken the U-shaped relationship between tool motivation-oriented corporate donations and innovation investment.

The theoretical contributions of this study are as follows. (1) This study extends the research paradigm on the relationship between corporate charitable donations and investment in innovation. This study constructed a research framework for classifying different types of charitable donation motivation by psychological motivation measurement methods, which provides a new entry point for a more systematic exploration of the relationship between charitable donations and innovation investment. That provides a new paradigm for answering the controversial " promoting or inhibiting" question in studying the relationship between corporate charitable donations and innovation investment and provides a valuable reference for studying corporate charitable donations from a diversified perspective. (2) By integrating resource dependence theory and agency theory, a new theoretical framework is provided to explore the nonlinear relationship between charitable donations to innovation investments. This study finds through literature review that corporate charitable donations affect the acquisition of external innovation resources and also profoundly affect the allocation of internal resources, while previous studies mainly explore the linear relationship between charitable donations and innovation investment based on a single theoretical perspective [1, 3, 4, 7, 15], and it is difficult to explore the complex relationship between the two in depth. Therefore, based on the integrated resource dependence theory and agency theory perspectives, this study explores the nonlinear relationship between charitable donations on innovation investment under different charitable donations motivations, respectively, and provides a more comprehensive theoretical framework for studying the mechanism of corporate charitable donations on innovation. (3) The findings enriches the knowledge in the relationship between corporate charitable donations and innovation investment. First, the findings of this study verify the "crowding-out effect" of charitable donations on corporate innovation investment, based on the view of scholars such as Shen Yi [4], and further confirm that the "crowding-out effect" and the "resource effect" exist in different ranges of the relationship between charitable donations and innovation investment for different donation motivations. This provides a microscopic knowledge base for subsequent studies to explore the matching of corporate donations with the environment and resources at the strategic level. Second, this study examines the moderating effect of state ownership on the relationship between corporate charitable donations and innovation investments. The findings validate the view of scholars such as Zhai H Y [56] and Wang L [50] that "government-enterprise relationship" exacerbates the "crowding out effect" of corporate charitable donations on innovation investment. And the study further found that for both types of charitable donations, state ownership exacerbates the multiple agency problem to varying degrees.

The practical implications of this study are as follows. (1) Under the background of the joint prevention and control of the COVID-19 in the whole society, enterprises cannot assume social responsibility simply through charitable donations as in the past. Economic organizations, including small and medium-sized enterprises and new enterprises, will face higher requirements and greater responsibilities given by society. Achieving the balance between economic goals and social responsibility goals is the key to whether enterprises can continue to adapt to social expectations. Therefore, enterprises should formulate a scientific management personnel selection system, strengthen executives’ supervision and management, and develop effective compensation incentive mechanisms to reduce the waste of agency problems on enterprise resource allocation. This study shows that state-owned property rights will further exacerbate the impact of agency problems on corporate charitable donations. Therefore, with the help of the current reform of mixed ownership of state-owned enterprises, the supervision and management departments of state-owned enterprises should constantly improve the management system of state-owned property enterprises, enhance the scientific and professional management, and break the shackles of bad bureaucracy in management. (2) It is necessary to strengthen government guidance on corporate philanthropy and improve the service role of government departments at all levels in the process. At present, there are various extreme donation behaviors such as “tool”, “speculation” or “rent-seeking” in corporate philanthropy. The reason is that there is an ‘agency’ problem between enterprises and the government and a lack of mechanism rules and corresponding regulatory measures in corporate philanthropy in China. Experience shows that government advocacy is still the biggest driver of the charity market. It is necessary to rely on coercive measures under formal systems such as laws and regulations and policy guidance. In this regard, the government must give full play to the guiding and service role of market economy, encourage responsible market activities and commercial behavior through the market mechanism, encourage and promote enterprises to fulfill their social responsibilities, build reasonable supportive and guiding measures, attach importance to and strengthen the construction of a suitable interaction mechanism between enterprises and society, and give full play to the supervision role of social norms mechanism, mobilize the public, the media, and other social forces under the informal system to participate in supervision, and gradually promote the sustainable development of corporate philanthropy and social responsibility to form a virtuous circle.

In addition, there are still some shortcomings in this study to be further added. Firstly, due to the use of second-hand data in the empirical analysis of this study, it is difficult to avoid the shortcomings and limitations of data errors. Therefore, on this basis, some conclusions may have errors that need further improvement. Secondly, due to the specificity of this research problem, the continuity of the research sample data obtained after screening is poor. We hope that we can obtain better quality sample data with the help of more scientific and practical means in the subsequent study to obtain more scientific and accurate research results. Thirdly, this study only explores the relationship between charitable donations and innovation investment in a relatively superficial way from the logic of resource acquisition and expenditure, with limited theoretical contribution and further theoretical depth to be explored in future studies.

## Supporting information

S1 Data (XLSX)
